# Re-imagining Ophthalmic Teaching and Education in Medical Programs: The ROTE Study

**DOI:** 10.7759/cureus.104997

**Published:** 2026-03-10

**Authors:** Zayn Al-Timimi, Josiah Romeo, Nayuta Yoshioka, Hamish Dunn, Jenny L Hepschke, Adrienne Torda, Lisa Jane Keay, Samuel D Browning

**Affiliations:** 1 School of Clinical Medicine, University of New South Wales, Coffs Harbour, AUS; 2 School of Optometry and Vision Science, University of New South Wales, Sydney, AUS; 3 Rural Clinical School, University of New South Wales, Port Macquarie, AUS; 4 Department of Ophthalmology, Save Sight Institute, University of Sydney, Sydney, AUS; 5 School of Clinical Medicine, University of New South Wales, Sydney, AUS

**Keywords:** andragogy, blended curriculum, ophthalmology, qualitative semi-structured interviews, undergraduate teaching

## Abstract

Background: Undergraduate ophthalmic teaching has been subject to a global decline as ophthalmology competes for space in an increasingly crowded medical curriculum. Rather than focusing solely on increasing teaching time, strategies are needed to optimise the use of existing time by ensuring that teaching is engaging and effectively improving students’ knowledge and confidence in ophthalmic skills. While the literature is rich with innovative teaching approaches for ophthalmology, the vast majority of these studies have been quantitative. This study seeks to fill this gap with a qualitative exploration of student perspectives and ideas for their own learning.

Methods: A mixed-method approach was used to evaluate perspectives, preferences, and ideas regarding ophthalmology teaching among senior medical students and recent graduates of the University of New South Wales, Australia. A quantitative questionnaire was used to assess participant ratings of teaching methods, while one-on-one semi-structured qualitative interviews and thematic analysis were employed to facilitate a richer exploration of participant perspectives.

Results: Quantitative data (n = 46) found that most participants (80%) received very little ophthalmology teaching time, and its effectiveness was considered poor by 57%. The two teaching methods considered least effective (didactic lectures and self-directed learning) were simultaneously the most common modes of teaching. Four themes (and 12 subthemes) emerged from the qualitative interviews (n = 12): a reason to learn, prioritising fundamentals, optimising existing learning opportunities, and re-imagining ophthalmic teaching.

Conclusions: The participants emphasised clinical relevance as a driver of learning in their approach to ophthalmic content, in alignment with adult learning theory (ALT). The participants perceived current ophthalmology teaching as insufficiently clinical, poorly structured, and inadequately integrated. They craved hands-on teaching and clinical exposure to spark their curiosity and intrinsic motivation to learn. A blended learning approach employing well-structured lectures and online modules with self-assessments, hands-on teaching in clinical settings, and competency-based assessments would represent students' and graduates' ideal re-imagining of the ophthalmic curriculum.

## Introduction

Vision impairment has an enormous impact on quality of life, with blindness considered the worst health complication imaginable by members of the public [1]. Along with primary ophthalmic disorders, a number of systemic diseases have potentially vision-threatening sequelae, and ocular manifestations may be the only signs of multiple potentially life-threatening conditions [2]. Thus, the ability to assess, interpret, and manage ophthalmic signs and conditions is essential to the training of safe medical practitioners [2]. These conditions comprise 2.1% of general practice consultations (equivalent to approximately two consultations per week) and between 1.5% and 11.8% of emergency department presentations [3-6]. Even when an ophthalmic complaint is not the reason for presentation, ocular examination should still be considered essential to any general health checkup [7]. For example, accurate fundoscopic examination for appropriate clinical presentations (such as visual disturbance, neurological disturbance, headache, and hypertensive urgency) can change management in up to 39% of cases [8]. Conversely, misdiagnosis can lead to significant, preventable adverse outcomes, including severe permanent vision loss [9].

Unfortunately, ophthalmic education has been subject to a precipitous decline in recent years as already crowded medical curricula, particularly undergraduate curricula, struggle under the pressure of expanding knowledge [10]. Ophthalmology has been deprioritised as core disciplines dominate increasingly crowded curricula [11]. Globally, average ophthalmic teaching time has decreased by approximately 20 hours per decade over the past two decades [2]. Three-quarters of medical students are not confident in their knowledge, and two-thirds are not confident in their skills [2]. This remains the case among junior doctors [12-14] and even senior clinicians, with 10% of general practitioners (GPs) in one survey admitting to being “scared stiff of eyes” [15]. The consequences of this decline in clinical confidence include under-recognition or incorrect treatment of serious eye conditions, in addition to over-referral of simple conditions [16].

There is an increasing volume of literature investigating teaching methods in ophthalmology [16-18]. Traditional didactic teaching is being replaced by problem-based learning, self-directed learning, and online teaching strategies [19]. The proliferation and implementation of new teaching techniques increased dramatically as educators devised innovative adaptations to the coronavirus pandemic, learning lasting lessons [20,21]. Many of these strategies have been grounded in adult learning theory (ALT), the foremost being andragogy [22,23]. Andragogy describes the adult learner as practical, relevance-oriented, goal-oriented, bringing life experience to learning experiences, and being equal with the educator [22]. This has also been accompanied by a paradigm shift across medical education toward student-centred teaching, which focuses on what the student does versus what the educator does or knows, treats students as active participants with high levels of choice over content/style/timing of learning, and views students rather than teachers as primary agents of learning [24]. However, in an environment where pedagogical principles advocate for adult learners to play an active role in guiding their education, the student perspective on ophthalmic teaching is largely unknown.

A detailed understanding of student perspectives on ophthalmic teaching is imperative to informing strategies to optimise student learning, engagement, and confidence. The majority of previous studies of student perspectives of ophthalmic teaching have relied on Likert-scale surveys or analysis of assessment results to assess satisfaction or efficacy of ophthalmic teaching [12,25-28]. None have used qualitative methods, which are needed to provide a more holistic insight into students’ points of view [29]. Accordingly, this study aimed to characterise students' experiences of ophthalmology teaching and explore their perspectives on the critical requirements for an effective curriculum using a predominantly qualitative, mixed-method approach.

## Materials and methods

We employed a mixed-methods design with a qualitative focus to explore senior medical students' and recent graduates’ views on ophthalmic education at a single Australian medical school (University of New South Wales (UNSW)). We aimed to identify learners’ issues with the current program and elicit their needs, goals, and objectives and their preferred educational strategies to meet steps one (problem identification), two (needs assessment), three (goals and objectives), and four (educational strategies) of Kern’s six-step approach to curriculum development [30], which was used as a conceptual framework to ground this study [31]. Quantitative questionnaires assessed the perceived efficacy of current teaching methods, while qualitative interviews provided deeper insights. The research team included two medical students (JR, SB) and academics with expertise in qualitative research, medical education, ophthalmology, optometry, and faculty leadership. Ethics approval was obtained from the UNSW (approval no. HC210140), with informed consent obtained from all participants, conducted in adherence to the Declaration of Helsinki, and reported in compliance with the Consolidated Criteria for Reporting Qualitative Studies (COREQ) statement [32].

Context

The UNSW Faculty of Medicine and Health offers a six-year undergraduate Bachelor of Medical Studies and Doctor of Medicine (BMedMD) program [33]. This program requires ongoing accreditation by the Australian Medical Council to ensure compliance with national standards for primary medical programs and is currently undergoing a major curriculum redesign. Simultaneous research evaluating the efficacy and implementation of asynchronous interactive ophthalmic teaching modules is also currently underway [34].

Participants

Students in the fifth or sixth year of the UNSW medical program or junior doctors who were recent graduates of the program (postgraduate years 1-3) were eligible. There were no further inclusion or exclusion criteria. Participants were invited to complete an expression of interest in the study via email, using year-level listservs supplied by course administrators and year-level social media groups (e.g., Facebook groups). 

Questionnaire

A quantitative questionnaire comprising multiple-choice and five-point Likert scale [35] questions was used to assess student preferences and experiences of a range of commonly employed and researched teaching methods. The full questionnaire is provided in Appendix A. Definitions were provided for the teaching methods assessed, and a consistent Likert scale was used whenever possible to aid objective analysis [36]. The questionnaire was administered online using Research Electronic Data Capture (REDCap) software [37].

Interviews

One-on-one interviews were conducted using a semi-structured interview guide, which enabled the researcher and participant to explore experiences that built understanding of the research topic. The full semi-structured interview guide is provided in Appendix B. The interview guide was designed to explore core tenets of ALT [38] and was piloted with several students not included in the final study. It remained open to further iteration, enabling integration of findings from successive interviews to better direct remaining interviews. A conversational approach adopting open-ended questions was used to foster unbiased data collection, with probing questions employed to encourage elaboration or clarification of concepts raised by participants when necessary. Interviews were conducted predominantly online (via Zoom video conferencing) for participant convenience and to enable multi-site recruitment [39]. Interviews were audio-recorded and transcribed verbatim by the interviewer. Recruitment continued until thematic saturation [40] was reached, operationally defined as the point where no new themes arose in three successive interviews.

Analysis

Questionnaire data were synthesised using descriptive statistics only. Interview transcripts underwent thematic analysis, initially adopting a deductive approach guided by ALT, then moving toward an inductive approach to maximise the capacity for analysis to remain data-near as the diversity of data increased [41]. The approach to thematic analysis employed was reflexive, in line with the Braun and Clarke approach [42]. Initially, two researchers (JR and SB) independently read and familiarised themselves with the first three transcripts before generating preliminary codes using NVivo V.12 (Lumivero Pty Ltd, Melbourne, Australia). These codes were then compared and discussed with the wider research team to develop a preliminary coding framework, with subsequent transcripts coded using this framework by one researcher (JR). The framework was iteratively refined through discussion and applied to subsequent transcripts. As additional transcripts were analysed, new codes were incorporated where necessary, and previously coded transcripts were revisited to ensure consistency. Regular meetings were held between investigators to review emerging themes and resolve discrepancies through consensus. Reflexivity was maintained throughout the analysis process by discussing how investigators’ backgrounds in ophthalmology, medical education, and student experience might influence the interpretation of the data. The diversity of educational and content experience of the research team ensured a holistic interpretation and served to enrich the analysis.

## Results

Questionnaire responses were received from 26 students and 20 graduates, spanning classes of 2018-2022. The composition and demographics of this cohort are provided in Table 1.

**Table 1:** Summary of study participant details. The questionnaire was completed by 46 participants, and semi-structured interviews were conducted with 12 participants. Proportions are provided for each of these study groups respectively, with age and sex detailed for the interview participants only. PGY: post-graduate year

	Questionnaire	Interviews		
	Participants (% of the total)	Participants (% of the total)	Median age (range)	Female participants (% of participants)
Students	26 (56.5%)	8 (66.7%)	23 (23-25)	3 (38%)
					Year 5	14 (30.4%)	4 (33.3%)
Year 6	12 (26.1%)	4 (33.3%)		
Graduates	20 (43.5%)	4 (33.3%)			24.5 (24-27)	2 (50%)
			PGY1	8 (17.4%)			2 (16.7%)
PGY2	8 (17.4%)	1 (8.3%)				
PGY3	4 (8.7%)	1 (3.3%)				
Total	46	12	24 (23-27)	5 (42%)		

**Figure 1:**
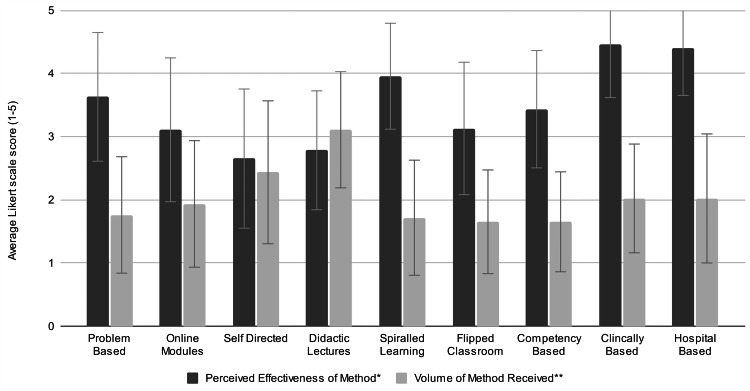
Comparison of average perceived effectiveness and volume received of teaching methods, with standard deviation error bars. *1 terrible, 2 poor, 3 average, 4 good, 5 ideal/optimal. **1 none of, 2 little of, 3 some of, 4 most of, 5 all of Image created by the authors with MS Excel (Microsoft Corp., USA)

The majority of participants (37 participants; 80.4%) reported “very little” time allocated to ophthalmology teaching, and 57% (26 participants) reported that the effectiveness of the ophthalmic teaching they received was either “poor” or “terrible”. Participants unanimously agreed (100%) that “improvements could be made to the teaching of ophthalmology”. Students reported most commonly receiving didactic lectures and self-directed learning for ophthalmology, and simultaneously also rated these as the least effective teaching methods (see Figure 1).

Clinical-based learning and hospital-based tutorials were considered the most effective teaching methods. Thematic saturation was reached and confirmed after 12 qualitative interviews. The mean interview duration was 26.2 minutes and ranged from 15.2 to 43.3 minutes. Four major themes relating to experiences, preferences, and recommendations for ophthalmic teaching were derived, with illustrative quotes presented in Table 2.

**Table 2:** Illustrative quotes from thematic analysis, including sub-themes.

1. A Reason to Learn	
Relevance to assessment. Relevance to future practice. Intrinsic curiosity.	a. “I think you'd be more motivated to learn things out of interest or passion if we weren't given barrier exams … at the end of the day all that matters is Biomed [5th year barrier exam] … I don't think ophthalmology comes up in our Biomed past cases or protocols, so … as long as you can know your ophthalmology as far as cranial nerves is concerned or diabetes is concerned or hypertension is concerned, that's all you need.” [Student 4, 25M]
	b. “I think in general for you to learn something you need to have a goal, so why are you learning it in the first place…but from a medical student point of view there is this burden to know a lot of stuff so as a student I always feel like I need to know something because of my exams rather than because I’m actually interested in it.” [Junior Doctor 1, 24M]
	c. “I'm going to be an intern so soon, I need to be able to answer questions that are basic… so that I can, in a nutshell, not look stupid in front of patients.” [Student 2, 23M]
	d. “It would be good to know exactly what things you need to do before you call the ophthal reg...just knowing those practical things and having that framework.” [Student 3, 24F]
	e. “Just being able to say the basic simple stuff that you should be able to do makes you look like less of a fool on the phone to a person who’s an expert and has spent years and years doing this thing...and it's nice to not look like a total fool.” [Junior Doctor 2, 27F]
	f. “To get into medicine, you have to be a pretty anxious high achiever who, when left to their own devices, makes use of their time; that's pretty well a prerequisite." [Student 4, 25M]
	g. “Interest in ophthalmology would be best fostered by having people exposed to ophthalmology.” [Student 4, 25M]
	h. “I've never been interested, thought about ophthalmology at all, which is probably just because we’ve never been given that stimulus.” [Student 5, 23M]
2. Prioritising Fundamentals	
Core content is drowning in a crowded curriculum. Clarity of learning outcomes and integration. Guidance from assessments and exposure.	a. “In general, medical school wasn’t a good experience for me because there's this burden on me to know a lot of stuff, and knowing that I’m not gonna go into internal medicine or general surgery, why do I have to know all of these things?” [Junior Doctor 1, 24M]
	b. “Ophthalmology is probably low down on that list, medical students talk a lot about what’s high-yield...and I think the amount of times you’re going to see an ophthalmic issue compared to say an acute coronary syndrome … it's probably much less.” [Student 1, 23M]
	c. “When you’re an intern, and when you’re a resident, you have to do emergency terms, you don’t get out of it, you need to be able to assess a patient with a red eye or someone who’s had some sort of blunt trauma to the face.” [Junior Doctor 2, 27F]
	d. “They need to have a clear framework, or clear things like clinical outcomes, what do we need you to know from this?” [Junior Doctor 1, 24M]
	e. “If you have single subject or single focused lectures that go for 6 minutes, they're much, much better with knowledge retainment and attention retainment.” [Student 4, 25M]
	f. “You just don't really know where things fit in, you don't really have a good framework for how to approach ophthal issues, and I think we don't really have a good understanding of how eye stuff links in with all the other systemic diseases.” [Student 3, 24F]
	g. “Anything that pops up in an exam; so if I know there's a past question or a past case in it, I'm gonna study it.” [Student 2, 23M]
	h. “Even if we had a couple of days in an ophthal clinic or something...then at least you've got something to anchor your study on and figure out what are the key things you need to know, and then you can go away and do that learning.” [Student 3, 24F]
3. Optimising Existing Learning Opportunities	
Balancing guidance and independence. Quality over quantity. The greatest teacher is experience.	a. “It's difficult to do a lot of self learning if you're given a lot of compulsory learning, just out of time and attention span, you don't really want to spend a couple of hours doing your own passion homework and then have to turn around and do something that you're forced to do for another couple of hours.” [Student 4, 25M]
	b. “I think self-directed learning is a bit of a waste of time, to be honest, especially for something like this that needs to be hands-on… I had issues when I was at university with the way that there were whole areas of the course that were not covered, and it was expected that I would teach myself.” [Junior Doctor 2, 27F]
	c. “The uni has such high expectations on students for our presentations… they mark you on engagement and how much text you use on your slide and how many slides and did you use visual aids and da da da, but then you look at some of our lecture slides, and they obviously violate every single rule.” [Student 2, 23M]
	d. “They're still useful [didactic lectures], it's just they need to be structured in such a way that it's not information overload” [Student 3, 24F]
	e. “I didn't learn anything from those 4 or 5 hour-long, detail-heavy lectures, because they were just slide shows enormous amounts of detail and technical terms, half of which I've never heard before” [Student 4, 25M]
	f. “They’re just telling us what to see, but none of them taught us how to use a slit lamp and how to actually see the signs ourselves, so that’s what I found not helpful.” [Junior Doctor 1, 24M]
	g. “It’s all well and good to learn it from a book, but I personally find that if I meet a patient with that disease process and talk to the patient and understand their experience, it’s a much more real thing to me and I make a much stronger memory.” [Junior Doctor 2, 27F]
	h. “When you play around with equipment, it’s really good, like a slit lamp, the whole thing, even the desk is part of the thing, moving it, sitting in it correctly, setting it up, and there's like a million knobs and each of them does a different thing.” [Student 2, 23M]
4. Re-imagining Ophthalmic Teaching	
Ideal timing and scope of teaching. Ideal teaching methods. Ideal assessment methods.	a. “I feel like there'd be more scope to do a little bit more in phase two, linking it in with the clinical side of things, I think that would be helpful, because otherwise you just get to phase 3 and you've done nothing.” [Student 3, 24F]
	b. “I think maybe they need to introduce it, maybe in concert with the lectures, if there was a clinical skills session on the basics of the ophthal exam, which would tie into some of the cranial nerve examination.” [Junior Doctor 2, 27F]
	c. “I feel like it should be delivered when you’re finishing your medical school so that it will stick with you more” [Doctor 1, 24M]
	d. “I wouldn't want anything too regular because it’s such a small part, there are so many competing interests, I wouldn't want ophthal to become a big thing.” [Student 2, 23M]
	e. “The lectures are equally as important because they provide the foundations, it’s like a briefing.” [Junior Doctor 1, 24M]
	f. “They [general practice sexual health modules] made you actually think about the cases and apply your clinical knowledge, and they didn’t try to reinvent the wheel, they weren’t trying to teach you an entire specialty in a 15-minute module.” [Student 1, 23M].
	g. “You can condense a lot of critical information quickly for people to do at their own pace and then evaluate them with quizzes.” [Student 1, 23M]
	h. “A really good adaptive tutorial is really nice because I find that everything is well organised...you can pack the information in your brain in an organised way.” [Student 2, 23M]
	i. “I don’t think it’s appropriate to be assessing ophthalmology in vivas or OSCEs simply because we’re not taught enough of it to be able to confidently manage it.” [Student 1, 23M]
	j. “Best case scenario, you're assessed as competent rather than being very well rote-learned, that's my best answer, so a short lesson with a short, fairly meaningless assessment afterwards, it doesn't need to count towards anything for med students to think that it's important.” [Student 4, 25M]
	k. “Maybe a logbook with a few more things that need to be ticked off, not necessarily more different procedures, but just to show you've done the same procedure on a number of occasions, just to ensure there is that clinical competence before you're going out to do it in the wild.” [Junior Doctor 3, 24M]

Theme 1: a reason to learn

The participants felt their learning needed to serve a purpose and identified their key reasons for learning were to pass assessments, become a competent doctor, or follow inherent interests. Teaching content needed to be relevant to at least one of these motivating factors for it to be learnt. 

Relevance to Assessment

The primacy of barrier exams was considered the core determinant of content relevance by many participants due to the anxiety these examinations evoked (Table 2, quote 1a). The reality and/or perception of limited representation of ophthalmic content in such examinations meant that the participants were inclined to de-prioritise ophthalmology relatively swiftly as they triaged other content more frequently or extensively examined as being more important (Table 2, quote 1b). The participants also suggested that more frequent but lower-stakes assessment tasks would better encourage learning. 

Relevance to Future Practice

Relevance to future practice represented a key extrinsic motivator for the participants to devote time to ophthalmic content, with the participants identifying a fear of feeling or being seen as incompetent by senior doctors or patients (Table 2, quotes 1c-1e).

Intrinsic Curiosity

The participants identified natural curiosity as a key intrinsic motivator for learning (Table 2, quote 1f) and felt this was best fostered through clinical exposure (Table 2, quote 1g). Accordingly, limited ophthalmic exposure was highlighted as an important reason for limited ophthalmic confidence. Without meaningful clinical exposure to ophthalmology, there was limited scope for this intrinsic interest to inspire the participants to want to understand ophthalmic content (Table 2, quote 1h).

Theme 2: prioritising fundamentals

The participants reported significant superfluous detail in their teaching, which was considered problematic given the limited ophthalmic teaching time. This was compounded by a lack of clear learning outcomes and integration with other areas of medicine. Most participants prioritised content and skills with relevance to emergency rotations during internship and/or the general practice setting. However, content known to be examinable was given supreme priority, including taking precedence over more clinically-relevant content.

Core Content Drowning in a Crowded Curriculum

The participants described the difficulties of studying a crowded curriculum, with high expectations for increasingly detailed knowledge in a broad range of specialty areas. The participants felt that the reality of medical specialisation meant it was not necessary to gain expert knowledge in all areas of medicine (Table 2, quote 2a). Accordingly, some participants de-prioritised ophthalmology (Table 2, quote 2b). While the participants felt an extensive knowledge of ophthalmology was beyond the scope of their curriculum pressures, they still pinpointed some areas that they felt warranted study, such as developing an approach to a patient with a red eye, assessing ocular trauma, understanding red flags, and developing basic proficiency with slit lamp examinations (Table 2, quote 2c).

Clarity of Learning Outcomes and Contextualisation

The participants identified clear learning outcomes as essential to providing clarity and harmony between student and educator expectations (Table 2, quote 2d). Without clearly defined learning outcomes, the participants felt that their study was less directed and that their learning was less effective. They desired focused learning activities, ideally delivered in short, manageable chunks able to be accessed asynchronously (Table 2, quote 2e). In addition, the participants lamented the siloing of ophthalmic content, which they felt was minimally integrated with broader teaching. This lack of contextualisation meant some participants reported a limited appreciation of the relevance of ophthalmic content to other areas of medicine (Table 2, quote 2f). 

Guidance From Assessments and Exposure

The participants reported their study was primarily guided by the content they could be examined on (Table 2, quote 2g). Resources that predicted assessment content, such as past questions or clinical cases, were well utilised in the study, and ophthalmology was not overlooked when included in these resources. The participants secondarily prioritised content they considered important for future practice, which they determined based on clinical experiences. Accordingly, multiple participants highlighted the importance of clinical exposure to ophthalmology as essential to guiding learning (Table 2, quote 2h). 

Theme 3: optimising existing learning opportunities

The participants found many of their ophthalmic learning activities ultimately unhelpful. They stressed the critical importance of a balance between self-directed and supported learning and strongly felt that quality was more important than quantity of ophthalmic teaching. Practical experiences with hands-on demonstrations were valued far above any other mode of learning.

Balancing Guidance and Independence

The participants reported often finding current compulsory ophthalmic teaching ineffective, with some feeling this time would have been more effectively utilised for self-directed learning (Table 2, quote 3a). However, the participants also stressed that there was a limit to their capacity for self-directed learning in an area as specialised as ophthalmology, especially ophthalmic examination skills for which they stressed the importance of dedicated, hands-on experiential learning (Table 2, quote 3b).

Quality Over Quantity

Regardless of the type of teaching method employed, the participants emphasised the importance of producing teaching materials to a higher standard (Table 2, quote 3c). The merit of lectures and tutorials was recognised by the participants, but they emphasised the need for lectures to be well-structured (Table 2, quote 3d) and criticised didactic lectures with excessive detail (Table 2, quote 3e).

The Greatest Teacher is Experience

The participants criticised theoretical teaching, which was unaccompanied by experiential teaching (Table 2, quote 3f). They reasoned that clinical exposure provided an opportunity to create stronger memories associated with exposure to real patients, which enabled greater understanding and retention of content (Table 2, quote 3g). The participants pointed to hands-on teaching as the best way to learn the unique technical aspects of ophthalmic examination equipment, particularly the slit lamp, through supervised practice and troubleshooting with peers (Table 2, quote 3h).

Theme 4: re-imagining ophthalmic teaching

The participants described specific ways in which they thought ophthalmic teaching could be delivered effectively. Suggestions related to the structure and timing of ophthalmic teaching, as well as teaching and assessment methods. Online modules were frequently mentioned as a favourable teaching method.

Ideal Timing and Scope of Teaching

The participants encouraged the introduction of clinical ophthalmology earlier in the course, alongside theoretical content, to help contextualise clinical relevance. They felt that integration of ophthalmology alongside other core areas of the curriculum, especially clinical skills teaching, may be an efficient way to ensure its inclusion (Table 2, quote 4a). For example, it was suggested that teaching of ophthalmic examination skills would fit well with the neurological examination (Table 2, quote 4b). The optimal timing for ophthalmic teaching was somewhat contentious, with some advocating for increased teaching in the middle of the program (Table 2, quote 4b) and others toward the end of the program (Table 2, quote 4c). However, in the context of a crowded curriculum, the participants still felt the scope of ophthalmic teaching should remain limited (Table 2, quote 4d), focusing on essential, clinically relevant information only.

Ideal Teaching Methods

The necessity for didactic lectures to introduce initial content was recognised (Table 2, quote 4e), but there was a strong desire for lectures to be more focused and engaging. The participants placed a high value on hands-on learning and case-based learning alongside lectures to integrate theoretical content with clinical reasoning and diagnostic skills. There was significant support for online modules as a favourable method of content delivery. The participants referred to experiences with adaptive modules in other areas of medicine and highlighted the strengths of such modules in delivering case-based learning, which tested clinical reasoning (Table 2, quote 4f). They appreciated the flexibility of module-based learning, and valued modules that included questions and quizzes to test learning and clinical reasoning (Table 2, quote 4g). Such modules were preferably short and able to be repeated, thereby representing useful tools for revision. Again, the participants pointed to the importance of structure, with a well-structured online module leading to well-structured and organised learning (Table 2, quote 4h).

Ideal Assessment Methods

The participants understood the importance of assessment as a means of ensuring learning and competency. However, they felt that extensive examination of ophthalmology in barrier examinations would be inappropriate, given the limited amount and efficacy of teaching received (Table 2, quote 4i). Instead, the participants felt competency-based assessments, following clinical placements and/or clinical skills teaching, would be more appropriate and would still be taken seriously (Table 2, quote 4j). A logbook of clinical skills to be signed off by a supervisor was suggested as a tangible method of assessing competency, including sign-offs on a minimum number of patients/sessions to achieve competency through repetition (Table 2, quote 4k).

## Discussion

**Figure 2:**
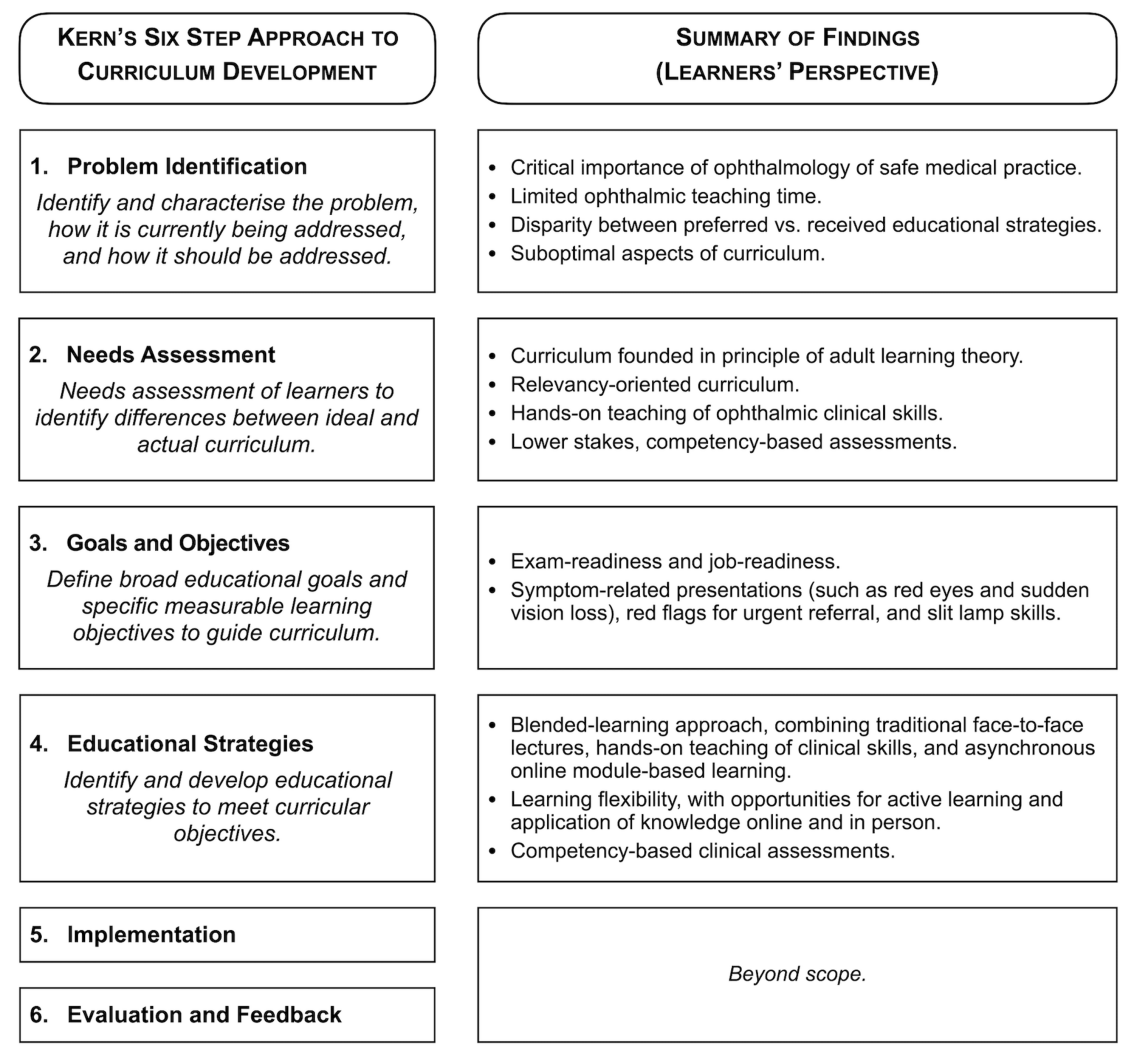
Learners’ perspectives regarding problems, needs, goals and objectives, and educational strategies for ideal ophthalmic teaching – steps 1-4 of Kern’s approach to curriculum development (authors' original work). Reference: [30]

This is the first qualitative study to explore medical students’ perspectives on ophthalmology education, reinforcing widespread concerns about its marginalisation in crowded curricula and offering student-driven solutions. The question of how to provide effective ophthalmic teaching in an increasingly crowded and time-constrained medical curriculum remains vexed. However, using Kern’s approach to curriculum development, this study offers student-driven insights for re-imagining ophthalmic teaching (Figure 2).

Problem identification

The participants reported limited ophthalmology teaching time, which is consistent with a global decline in the length of ophthalmic education in medical schools [2]. They identified multiple suboptimal aspects of their current program, including: excessive detail; excessive length; unfocused content with ambiguous learning objectives; insufficient focus on clinical relevance; poor integration with other areas of medicine; predominance of unengaging didactic teaching methods; insufficient ‘hands-on’ teaching; and lack of clinical exposure. Furthermore, the teaching methods which the participants found least effective - didactic lectures and self-directed learning - were those they received most frequently. 

Learners’ needs

**Figure 3:**
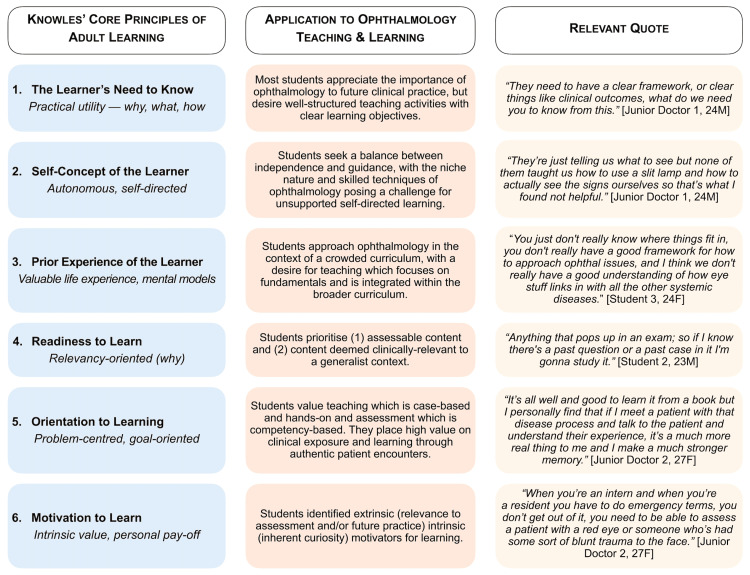
Conceptualisation of participant perspectives and preferences for ophthalmology teaching within Knowles’ core principles of adult learning theory (authors' original work). Reference: [43]

Thematic analysis highlighted how learners in this cohort were discriminating in their learning needs and preferences, in alignment with Knowles’ principles [43] of ALT, as conceptualised in Figure 3. The participants sought relevance, autonomy, and practical application - hallmarks of adult learners. Paramount in Knowles’ ALT framework is the learners’ ‘need to know’, with the participants in this study being strongly relevance-oriented and driven by intrinsic and extrinsic motivators. The participants viewed studying medicine as necessitating high intrinsic motivation, which is supported by research showing intrinsic motivation to be a predictor of good academic outcomes [44]. Assessment was a key extrinsic motivator, with students prioritising content linked to assessments. However, the participants criticised high-stakes assessment without adequate teaching. They advocated instead for low-stakes, competency-based assessments embedded in clinical experiences - preserving motivation without undermining autonomy. The participants also emphasised the need for hands-on teaching of ophthalmic clinical skills, which they did not believe could be effectively taught in the online environment or through self-directed learning.

Learners’ goals and objectives

The participants reported exam-readiness and job-readiness as critical goals for their ophthalmology teaching to accomplish. For the former, the participants independently emphasised the need for clear educator expectations for teaching activities, reporting insufficient understanding of their educators’ goals for their ophthalmology learning. This points to a need for pre-defined learning objectives to serve as a basis for constructive alignment of curricula; ensuring teaching and assessments are developed to address and appropriately reflect pre-defined learning goals [45,46]. Without clear learning objectives, there is limited scope for educator accountability or the measurability of student learning. 

With regard to job readiness, the participants considered symptom-related presentations (such as red eyes and sudden vision loss), red flags for urgent referral, and slit lamp skills as being most relevant. Benchmark curricula, such as the International Council of Ophthalmology’s curriculum for medical students [47], represent an ideal set of learning objectives to strive toward. However, students’ desire for content relevant to generalist practice is an important consideration and can be effectively facilitated through the involvement of generalist stakeholders (GPs, optometrists, students) in defining learning objectives, timing, length, and content of undergraduate ophthalmology [16,48,49]. 

Learners’ preferred educational strategies

The participants strongly preferred and uniformly lamented the lack of in-person, practical teaching and clinical exposure to ophthalmology. This disparity between preference for and exposure to clinical-based learning and hospital-based tutorials/workshops as educational strategies mirrors Zhang et al.’s larger survey of over 800 medical students and junior medical officers [12] and may explain repeated findings of low confidence in ophthalmic knowledge and skills among junior doctors [2,12-14].

Rather than focusing solely on increasing teaching time, strategies are needed to better optimise the limited time allotted to ophthalmology [17]. Many of the curriculum issues identified by the participants in this study stem from a lack of student-centredness in teaching approaches, with the current curriculum representing teacher-centred teaching - focusing on what the educator does or knows, and treating students as passive participants with limited choice over content delivery [24]. However, the literature is rich with student-centred techniques, which have been successfully employed in ophthalmic education, including “flipped classroom” models [28,50,51], case-based learning [50,51], team-based activities [25,52], peer-assisted activities [53], virtual reality simulations [54] and virtual clinics [55], and online modules [34,56]. The latter was particularly endorsed by the participants in this study, who pointed to positive experiences with online modules in other specialties. The participants favoured short, case-based modules that supported clinical reasoning and flexibility. A well-structured asynchronous online module was seen as enabling flexibility, student control over learning, improvement of clinical reasoning through case-based learning, and on-demand self-assessment. This accords with Petrarca et al.’s randomised controlled crossover study of third-year medical students, which demonstrated significant improvements in examination performance and satisfaction with asynchronous online modules compared to traditional lectures [56].

**Figure 4:**
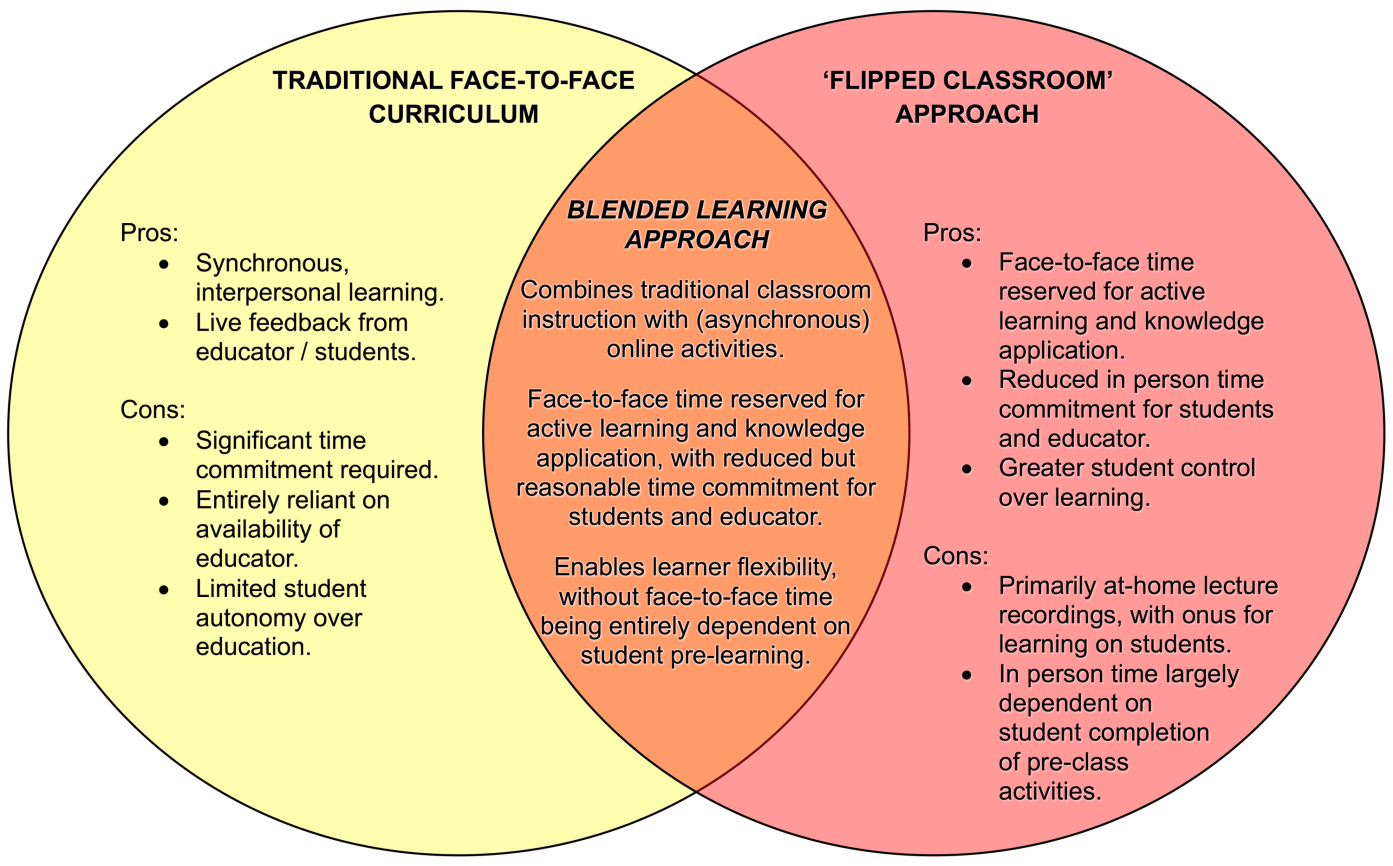
Features of a blended learning approach to medical education, compared to traditional and flipped classroom approaches (authors' original work). Reference: [57] Image created by the authors with iOS Pages (Apple, USA)

The participants also saw a role for traditional face-to-face teaching to deliver foundational content and outline learning goals, in conjunction with module-based learning. This desire for balance between guidance and independence articulated by the participants in this study would most likely be satisfied by a blended learning approach (Figure 4), combining in-person teaching with (typically asynchronous) online teaching and providing opportunities for active learning and knowledge application in both environments [57]. This avoids the burden of pre-learning of a flipped classroom approach, which, in the context of an overburdened medical curriculum in which ophthalmology is already fighting for space [11,58-60], represents an unreasonable expectation on students. Blended learning also provides greater flexibility and requires less time allocation than a traditional face-to-face curriculum, while still facilitating critical face-to-face time. Curricula with no face-to-face time, such as the synchronous online curricula adopted during the COVID-19 pandemic, have been shown to lead to significant student dissatisfaction (including difficulties understanding educator expectations), poorer staff motivation of students and provision of feedback, and poorer development of problem-solving skills [61]. Doyle et al. have demonstrated superiority of the blended approach over a synchronous online flipped classroom approach in ophthalmic teaching, with students in the blended learning group reporting increased satisfaction, clearer understanding of educator expectations, and increased subjective improvement in analytical and teamwork skills [27]. 

Finally, in terms of assessments, the participants in this study felt competency-based assessments, following clinical placements and/or clinical skills teaching, represented the most appropriate means of assessing ophthalmic knowledge and skills. In contrast to barrier assessments, this style of assessment enables more frequent, lower-stakes evaluation of learning. This approach has been successfully implemented by Succar et al. at the University of Sydney through a logbook with a minimum number of cases, a range of disease exposures, and skill competencies to be signed off by the supervisor [60]. Their curriculum (delivering content through lectures, self-directed learning, and clinical tutorials) and logbook were shown to increase learning, including retained knowledge at 12 months, and student satisfaction [60]. However, it would be important to ensure the scope of such a logbook was commensurate with the length of clinical placement - insufficient logbook items would not be meaningful, whereas excessive items may risk prioritisation of the logbook over authentic engagement with the placement itself.

Implications and applications

Although conducted within a single Australian undergraduate medical program, this study’s findings resonate with international reports describing declining ophthalmic teaching time across the United Kingdom, North America, Europe, and parts of Asia [2]. The themes identified - limited curricular space, student preference for clinical relevance, and dissatisfaction with didactic teaching - appear to reflect structural pressures common to modern medical education globally.

In countries with strong primary care systems (e.g., Australia, UK, Canada), undergraduate ophthalmic competence is particularly important given the frequency of ophthalmic presentations in general practice and emergency departments. Conversely, in systems with direct specialist access, foundational recognition of urgent pathology remains critical to safe triage. The blended, modular approach proposed here may be especially applicable across diverse resource settings. Short asynchronous modules require relatively modest infrastructure and can be disseminated at scale, including in low-resource settings where exposure to ophthalmology placements may be limited. Meanwhile, competency-based clinical sign-offs can be adapted to local contexts, whether supervised by ophthalmologists, general practitioners, or trained allied eye health professionals.

Strengths and limitations

This study’s strengths include its clear rationale, appropriate mixed-methods design, theoretical grounding, and transparent qualitative analysis. This study has focused on qualitative interviews to capture the richness and diversity of student perspectives not typically accessible by quantitative methods. Although the cohort was modest, it achieved thematic saturation and aligns with qualitative research standards [40]. However, the reliability of the quantitative survey results is limited by its small and selective sample, and the minimal demographic data collected has limited the better characterisation of the study cohort. Accordingly, the quantitative findings should primarily be regarded as defining the cohort and positioning the qualitative findings. The potential for reviewer bias must also be recognised as a limitation. Insofar as is possible, this has been mitigated through the reflexive analysis approach adopted, acknowledging the investigators' backgrounds and leveraging the diversity of the research team to provide holistic data interpretation. As with most qualitative research, the aim of this study is to generate analytical insight and deepen understanding of the phenomenon under investigation rather than to produce definitive or universally generalisable conclusions. As this study was conducted within a single Australian undergraduate medical program, the recommendations should be interpreted as context-informed insights rather than universally prescriptive solutions, and should be adapted to local curricular structures. While generalisability may be limited, this study's findings appear to resonate with the international literature.

## Conclusions

Students in this study were highly relevance-driven, preferring practical, clinically contextualised teaching aligned with assessments and future practice. However, they commonly encountered unfocused, didactic content delivered in isolation from clinical application - a disconnect that undermines confidence and learning. Four potential strategies to inform curriculum design emerge from this study: (1) identification of core ophthalmic competencies aligned with generalist practice (e.g., assessment of the red eye, recognition of sudden vision loss, safe use of the slit lamp, and identification of urgent referral red flags); (2) short, asynchronous case-based modules, incorporating adaptive questioning and immediate feedback; (3) prioritisation of face-to-face teaching time for hands-on examination skills and supervised practice, and; (4) competency-based sign-offs during clinical placements to ensure essential skill acquisition without overburdening high-stakes assessments. Critically, these changes do not require expansion of total teaching hours. Rather, they represent redistribution toward higher-value learning activities through a blended learning approach, better constructive alignment, and clearer accountability for skill acquisition. By aligning teaching methods with adult learning principles and student preferences, ophthalmic education can be revitalised and limited curricular time optimised.

## Appendices

Appendix A 

**Table 3:** Quantiative questionnaire

Quantitative survey (questionnaire)		
1. I am a:		☐ 5th year medical student ☐ 6th year medical student ☐ PGY1 Doctor ☐ PGY2 Doctor ☐ PGY3 Doctor
2. In your experience, how much teaching time was/is allocated for ophthalmology education and training at UNSW Medical School?		☐ No teaching time ☐ Very little time ☐ Appropriate time ☐ More than enough time ☐ Too much time
3. Of the ophthalmic teaching that you did receive at university, rate its effectiveness for fostering learning and retention of important ophthalmic skills and knowledge:		☐ Not applicable ☐ Terrible ☐ Poor ☐ Average ☐ Good ☐ Ideal/optimal
4. How effective do you think each of the following methods WOULD BE for taching ophthalmology in the UNSW Medicine course?	a. PROBLEM-BASED LEARNING — A student centered approach in which students learn about a subject by working in groups to solve an open-ended problem. The problem is what drives the motivation and the learning.	☐ Terrible ☐ Poor ☐ Average ☐ Good ☐ Ideal/optimal
	b. ONLINE MODULES — A teaching method which utilises interactive online courses which both deliver content and assess competency simultaneously.	☐ Terrible ☐ Poor ☐ Average ☐ Good ☐ Ideal/optimal
	c. SELF-DIRECTED LEARNING — Where students are directed to the topics and content they are required to learn and are given autonomy over how and when they learn the content and skills.	☐ Terrible ☐ Poor ☐ Average ☐ Good ☐ Ideal/optimal
	d. DIDACTIC LECTURES — a teacher-centred method of education where the teacher gives instructions to the students and the students are mostly passive listeners.	☐ Terrible ☐ Poor ☐ Average ☐ Good ☐ Ideal/optimal
	e. SPIRALLED LEARNING — A teaching method where skills and content are reviewed and encountered repetitively, based on the premise that student learns more about a subject each time the subject is reviewed.	☐ Terrible ☐ Poor ☐ Average ☐ Good ☐ Ideal/optimal
	f. FLIPPED CLASSROOM — Where students complete readings prior to the class, where in-class they then spend more time on collaborative activities that clarify concepts and contextualise knowledge through application.	☐ Terrible ☐ Poor ☐ Average ☐ Good ☐ Ideal/optimal
	g. COMPETENCY-BASED LEARNING — A system of instruction, assessment, grading, and academic reporting that are based on students demonstrating that they have learned the knowledge and skills they are expected to learn as they progress through their education.	☐ Terrible ☐ Poor ☐ Average ☐ Good ☐ Ideal/optimal
	h. CLINICALLY-BASED LEARNING — Where stuC14:C16dents attend ophthalmology outpatient department or emergency department and receive tuition in a clinically simulated environment.	☐ Terrible ☐ Poor ☐ Average ☐ Good ☐ Ideal/optimal
	i. HOSPITAL-BASED TUTORIALS/WORKSHOPS — Where students receive tutorials from clinicians in the environment the skills/knowledge is utilised.	☐ Terrible ☐ Poor ☐ Average ☐ Good ☐ Ideal/optimal
	j. OTHER METHODS (OPTIONAL) — Use this optional space for describing any other teaching methods you received (can be left blank).	☐ Terrible ☐ Poor ☐ Average ☐ Good ☐ Ideal/optimal
5. How much of the ophthalmic teaching at medical school WAS DELIVERED using these methods?	a. PROBLEM-BASED LEARNING — A student centered approach in which students learn about a subject by working in groups to solve an open-ended problem. The problem is what drives the motivation and the learning.	☐ None of ☐ Little of ☐ Some of ☐ Most of ☐ All of
	b. ONLINE MODULES — A teaching method which utilises interactive online courses which both deliver content and assess competency simultaneously.	☐ None of ☐ Little of ☐ Some of ☐ Most of ☐ All of
	c. SELF-DIRECTED LEARNING — Where students are directed to the topics and content they are required to learn and are given autonomy over how and when they learn the content and skills.	☐ None of ☐ Little of ☐ Some of ☐ Most of ☐ All of
	d. DIDACTIC LECTURES — a teacher-centred method of education where the teacher gives instructions to the students and the students are mostly passive listeners.	☐ None of ☐ Little of ☐ Some of ☐ Most of ☐ All of
	e. SPIRALLED LEARNING — A teaching method where skills and content are reviewed and encountered repetitively, based on the premise that student learns more about a subject each time the subject is reviewed.	☐ None of ☐ Little of ☐ Some of ☐ Most of ☐ All of
	f. FLIPPED CLASSROOM — Where students complete readings prior to the class, where in-class they then spend more time on collaborative activities that clarify concepts and contextualise knowledge through application.	☐ None of ☐ Little of ☐ Some of ☐ Most of ☐ All of
	g. COMPETENCY-BASED LEARNING — A system of instruction, assessment, grading, and academic reporting that are based on students demonstrating that they have learned the knowledge and skills they are expected to learn as they progress through their education.	☐ None of ☐ Little of ☐ Some of ☐ Most of ☐ All of
	h. CLINICALLY-BASED LEARNING — Where stuC14:C16dents attend ophthalmology outpatient department or emergency department and receive tuition in a clinically simulated environment.	☐ None of ☐ Little of ☐ Some of ☐ Most of ☐ All of
	i. HOSPITAL-BASED TUTORIALS/WORKSHOPS — Where students receive tutorials from clinicians in the environment the skills/knowledge is utilised.	☐ None of ☐ Little of ☐ Some of ☐ Most of ☐ All of
	j. OTHER METHODS (OPTIONAL) — Use this optional space for describing any other teaching methods you received (can be left blank).	☐ None of ☐ Little of ☐ Some of ☐ Most of ☐ All of
6. Do you feel that improvements could be made to the teaching of ophthalmology clinical knowledge and skills at medical school?		☐ Yes ☐ No

Appendix B 

**Table 4:** Qualitative interview guide

Qualitative interview guide		
Introduction	• Welcome and thanks.	
	• Name of interviewer, explain purpose of research, what this interview will add.	
	• Gain informed verbal consent.	
Previous Experiences	1. Can you tell me about your experience of ophthalmic education whilst at university?	Prompts: What were the positive elements? What were the not-so-good elements
Learning Styles	2. Can you tell me about the program methods, or teaching styles from medical school that have benefited your learning the most?	Prompts: What is it that makes [insert method] good? What methods are not so good? Why is that? Are there any other methods that have been beneficial?	
Teaching Styles and Delivery	3. What do you think are the most important education strategies for effective ophthalmic teaching?	Prompts: Why is this important for ophthalmic education in particular? Are there any barriers to ophthalmic education? How could these be removed? Are there any teaching methods that are to be avoided in ophthalmic education?
	4a. Can you tell me about what you think are the best ways that ophthalmic content could be delivered/implemented?	Prompts: Why would this [insert participants response] be beneficial to ophthalmic education? Why not other methods? How should the university approach this across the course of the medical program?
	4b. Can you tell me about what you think are the best ways that ophthalmic skills could be delivered/implemented?	
Curriculum Goals	5. What should be the ultimate aims of an effective ophthalmic education program at university?	Prompts: What should be the intended product of ophthalmic education during medical school? Why so? How do the overarching aims contribute to this?
Assessments	From your experience, how should this content be assessed by the university?	Prompts: Do you think you have been adequately assessed on ophthalmology by your medical program? What assessment styles do you find most conducive to learning? How should these be integrated into the program?
